# The Phytochemical Composition and Antioxidant Activity of *Matricaria recutita* Blossoms and *Zingiber officinale* Rhizome Ethanol Extracts

**DOI:** 10.3390/nu17010005

**Published:** 2024-12-24

**Authors:** Anca Elena But, Raluca Maria Pop, Georg Friedrich Binsfeld, Floricuța Ranga, Meda Sandra Orăsan, Andra Diana Cecan, Iulia Ioana Morar, Elisabeta Ioana Chera, Teodora Irina Bonci, Lia Oxana Usatiuc, Mădălina Țicolea, Florinela Adriana Cătoi, Alina Elena Pârvu, Mircea Constantin Dinu Ghergie

**Affiliations:** 1 Pathophysiology, Department of Morphofunctional Sciences, Faculty of Medicine, “Iuliu Hațieganu” University of Medicine and Pharmacy Cluj-Napoca, 400012 Cluj-Napoca, Romania; ancabut@umfcluj.ro (A.E.B.); orasan.meda@umfcluj.ro (M.S.O.); andra.cecan@umfcluj.ro (A.D.C.); iulia.morar@umfcluj.ro (I.I.M.); chera.elisabeta@umfcluj.ro (E.I.C.); adam.teodora@umfcluj.ro (T.I.B.); lia.usatiuc@umfcluj.ro (L.O.U.); madalinaticolea@umfcluj.ro (M.Ț.); adriana.catoi@umfcluj.ro (F.A.C.); parvualinaelena@umfcluj.ro (A.E.P.); 2 Pharmacology, Toxicology and Clinical Pharmacology, Department 2—Functional Sciences, Faculty of Medicine, “Iuliu Hațieganu” University of Medicine and Pharmacy Cluj-Napoca, 400012 Cluj-Napoca, Romania; 3 Psychiatry Department, Asklepios Westklinikum, Suurheid 20, 22559 Hamburg, Germany; georg.binsfeld@aol.de; 4 Food Science and Technology, Department of Food Science, University of Agricultural Science and Veterinary Medicine Cluj-Napoca, Calea Mănăștur, No 3-5, 400372 Cluj-Napoca, Romania; florica.ranga@usamvcluj.ro; 5 Orthodontics, Department of Conservative Odontology, Faculty of Dental Medicine, “Iuliu Hațieganu” University of Medicine and Pharmacy Cluj-Napoca, 400012 Cluj-Napoca, Romania; ghergie.mircea@umfcluj.ro

**Keywords:** *Matricaria recutita* blossoms, *Zingiber officinale* rhizomes, inflammation, oxidative stress, antioxidant

## Abstract

Background: Inflammation-induced oxidative stress is a pathophysiological mechanism of inflammatory diseases. Treatments targeting oxidative stress can reduce inflammatory tissue damage. Objectives: This study aimed to conduct phytochemical analysis and evaluate the antioxidant effects of the hydroalcoholic extract of *Matricaria recutita* blossoms (*M. recutita*) and *Zingiber officinale* rhizomes (*Z. officinale*). Materials and Methods: The phytochemical analysis was carried out by measuring the total polyphenol content, total flavonoid content, and polyphenolic compounds’ HPLC-ESI MS. The antioxidant activity was evaluated in vitro through H_2_O_2_ DPPH, FRAP, and NO scavenging assays. An in vivo experiment was performed on rats with turpentine oil-induced acute inflammation. Treatments were administrated orally for 10 days, with three dilutions of each extract (100%, 50%, 25%), and compared to the CONTROL, inflammation, Diclofenac, and Trolox groups. In vivo, the antioxidant activity was evaluated by measuring the total antioxidant capacity (TAC), total oxidative status (TOS), oxidative stress index (OSI), malondialdehyde (MDA), nitric oxide (NO), advanced oxidation protein products (AOPP), and total thiols (SH). Results: The phytochemical analysis found a high content of phenolic compounds in both extracts, and the in vitro antioxidant activity was significant. In vivo, *M. recutita* and *Z. officinale* extracts proved to be effective in increasing TAC and lowering oxidative stress markers, respectively, the TOS, OSI, MDA, and NO levels. The effects were dose-dependent, with the lower concentrations being more efficient antioxidants. *Matricaria recutita* and *Z. officinale* extract effects were as good as those of trolox and diclofenac. Conclusions: Treatment with *M. recutita* and *Z. officinale* alleviated inflammation-induced oxidative stress. These findings suggest that *M. recutita* and *Z. officinale* extracts could be a promising adjuvant antioxidant therapy in inflammatory diseases.

## 1. Introduction

Nowadays, although pharmaceutical remedies have evolved a lot, people tend to look more and more for naturopathic treatments, which have been used for thousands of years. That is why there is increasing interest in studying the anti-oxidant and anti-inflammatory effects of certain plants and natural extracts.

Dating back to ancient Egypt, diseases were cured using plant infusions and different oils made of plant parts. This idea withstood time. Thus, people still use plants as a basis for prophylactic and therapeutic treatments for different types of ailments, such as stomach pain, headaches, common cold symptoms, and myalgia [1].

As tea consumption has seen a significant increase in European countries in the last 10 years, phytotherapy has become an adjunct method to conventional medicine, more and more frequently used [2].

Inspired by that idea, this article aimed to highlight the efficacy of chamomile (*Matricaria recutita (M. recutita)*) and ginger tea (*Zingiber Officinale* (*Z. Officinale*)) as antioxidants and anti-inflammatory species that may yield medical relevance in treatment plans.

Inflammation is a non-specific complex response to any foreign pathogen or trauma, such as a physical injury, chemical exposure, or viral, bacterial, or fungal pathogens [3]. It is an adaptable response, of variable intensity. It depends on the type and contact time to the causative pathogen and may vary in intensity and duration, depending on the etiology of the offending mechanism. Inflammation in itself is not a disease, but a response to a foreign offender.

When inflammation persists, chronic inflammation develops. Recent studies maintain that chronic inflammation can not only be the starting point of carcinogenesis, but also plays an important role in the development and proliferation of cancer [4]. There are also many other serious diseases associated with inflammation, including heart disease, gastrointestinal diseases, and autoimmune diseases, many of which have increased mortality rates.

Inflammation can be caused by endogenous or exogenous pathogens, with the capacity to stimulate inflammatory cells and initiate inflammatory signaling pathways, primarily the MAPK, NF-κB, and JAK-STAT pathways [5].

Exogenous inflammation is induced via molecular patterns, associated with pathogens (PAMPs), which are highly specific and unique to microbial structures. They are bound by pattern recognition receptors (e.g., Toll-like receptors (TLRs), C-type lectin receptors (CLRs), RIG-I-like receptors (RLRs), NOD-like receptors (NLRs)). Extracellular TLRs first connect to lipids, pathogen-specific proteins, and carbohydrates (e.g., flagellin), which are bound to the pathogen’s surface.

Endogenous TLRs react differently, first connecting to the nucleoid acids that are in the endosomes after the process of phagocytosis. The process of a PAMP attaching to a TLR triggers the polymerization of the receptor [6]. After macrophages recognize the PAMP or DAMP (damage-associated molecular patterns), they activate NF-kB, which is a transcription factor, consequently increasing the expression of IL-1ß, IL-6, IL-1R, IL-6R, TNFR, and other inflammatory mediators, which are pro-inflammatory cytokines [7]. The inflammatory process also leads to the emergence of reactive oxygen species (ROS), which influence the progression of inflammation by acting as both a signaling molecule and a mediator [8].

The imbalance between antioxidants and oxidants (ROS and RNS), produced when inflammation is persistent and intense, causes oxidative stress [9], thus exacerbating the disease process.

Chamomile (*Matricaria chamomilla* or *M. recutita*), a member of the *Asteraceae* family, is among the most widely used plants with proven therapeutic effects in traditional and natural medicine. Its medicinal and cosmetic applications stem from a rich composition of active compounds, including phenolic acids (chlorogenic and caffeic acids), flavonoids (apigenin, luteolin, quercetin, and rutin), and coumarins (herniarin and umbelliferone). Over 36 flavonoids, 30 terpenoids, and 50 additional bioactive compounds have been identified in chamomile [10], suggesting its pharmacological potential. Studies have demonstrated its antibacterial, anti-inflammatory, sleep-enhancing, and depression-relieving properties, with flavonoids playing a significant role in its anti-inflammatory effects [5,11,12].

Ginger (*Z. officinale*), originating from Southeast Asia, is a herbaceous plant of the *Zingiberaceae* family. Its active components are categorized as volatiles (e.g., sesquiterpenes and monoterpenoids, responsible for its aroma and spiciness) and non-volatiles (e.g., gingerols, shogaols, and zingerone) [13]. Compounds like 6-shogaol exhibit potent antioxidant and anti-inflammatory effects by scavenging free radicals and inhibiting enzymes such as 5-lipoxygenase and prostaglandin synthetase [14]. Ginger also protects against ethanol-induced liver toxicity and reduces inflammatory cytokines, such as TNF-α and interleukins, with antioxidant effects comparable to ascorbic acid [15,16].

Ginger and chamomile are two of the most commonly used plants in alternative medicine. Studies have shown that almost 80% of the population tends to use traditional medicine as primary health care, but herbal extracts and their active components are often required as an adjuvant for this therapy, especially for preventive measures [17].

This study intended to contribute to the current literature and assess the efficacy of *M. recutita* and *Z. officinale* as antioxidants and anti-inflammatory species of potential medical relevance.

## 2. Materials and Methods

### 2.1. Chemicals

N-(1-Naphthyl) ethylenediamine dihydrochloride (NEDD), ferrous ammonium sulphate, sulphanilamide(SULF), sulphuric acid, hydrochloride acid, glycerol, trichloroacetic acid (TCA), Folin–Ciocalteu reagent, vanadium (III) chloride, trolox (6-hydroxy-2,5,7,8- tetramethylchroman-2-carboxylic acid), methanol, diethylether, xylenol orange [o-cresosulfonphthalein-3,3-bis (sodiummethyliminodiacetate)], trupentine oil, ortho dianisidine dihydrochloride (3-3′-dimethoxybenzidine), hydrogen peroxide, and complete Freud`s adjuvant were used in this study. They were procured from Sigma-Aldrich (Taufkirchen, Germany) and Merck (Darmstadt, Germany). The alcoholic solutions were supplied by the Physical and Chemical Testing Laboratory SC Prodvinalco SA (Cluj-Napoca, Romania).

### 2.2. Plant Material and Extract Preparation

The blossoms of *M. recutita* and *Z. officinale* rhizomes were purchased at a market in Cluj-Napoca.

Using a cold percolation process, the chamomile and ginger materials were extracted for three days at room temperature using a 60% ethanol (Merck, Bucureşti, Romania) concentration in the Mycology laboratory of “Babeş-Bolyai” University, Cluj-Napoca, Romania [18].

### 2.3. Phytochemical Analysis

#### 2.3.1. Total Polyphenol Content

*Matricaria recutita* and *Z. officinale* total polyphenol contents were spectrophotometrically determined using the Folin–Ciocâlteu method, with some modifications. To summarize, 2 mL of the ethanol extract from each plant was diluted 25 times and then mixed with 10.0 mL of distilled water and 1 mL of the Folin–Ciocâlteu reagent. The mixture was further diluted to 25 mL with a 290 g/L sodium carbonate solution. After incubating in the dark for 30 min, the absorbance was measured at 760 nm using a JASCO V-530 UV-vis spectrophotometer (Jasco International Co., Ltd., Tokyo, Japan). All TPC measurements were conducted three times, separately, under the same conditions, to certify the reliability and accuracy of our results. TPC values are reported as gallic acid equivalents (GAEs) (R^2^ = 0.999), mg GAE/g dry weight of herbal material [19].

#### 2.3.2. Total Flavonoid Content

The flavonoids’ overall content was quantified by measuring the total flavonoid content (TFC) of *M. recutita* and *Z. officinale*. This was assessed by combining 5 mL of the plant extract with 5.0 mL of sodium acetate (100 g/L) and 3.0 mL of aluminum chloride (25 g/L). The mixture obtained was then diluted to 25 mL with methanol in a standardized measuring flask. The absorbance was recorded at 430 nm, and the procedure was repeated three times. The TFC was calculated as quercetin equivalents (QE) (R^2^ = 0.999), expressed in mg QE per gram of dry weight (d.w.), of the herbal material [20].

#### 2.3.3. High-Performance Liquid Chromatography Coupled with Electrospray Ionization Mass Spectrometry Analysis (HPLC-DAD-ESI^+^)

The assessment for this part of our study was performed using an Agilent 1200 HPLC system, which incorporates a quaternary pump, autosampler, solvent degasser, and UV-Vis photodiode array detector (DAD). This system was linked to an Agilent 6110 single quadrupole mass spectrometer (MS). Compound separation was achieved using a Kinetex XB C18 column (4.6 × 150 mm, 5 μm particles; Phenomenex, Torrane, CA, USA). The mobile phases consisted of (A) water + 0.1% acetic acid and (B) acetonitrile with 0.1% acetic acid, following the gradient outlined below, over a 30 min period at a temperature of 25 °C, and a flow rate of 0.5 mL/min. Spectral data were recorded between 200 and 600 nm for all peaks, with chromatograms captured at wavelengths of λ = 280 and 340 nm. For mass spectrometry, the positive ESI ionization mode was used in full scan, with the following parameters: capillary voltage of 3000 V, a temperature of 350 °C, 7 L/min nitrogen flow rate, and a mass-to-charge (m/z) range of 120–1200. Data acquisition and analysis were conducted using Agilent Chem Station software, version B.02.01-SR2 [21].

Acetonitrile of HPLC grade was obtained from Merck (Germany), and the Direct-Q UV system from Millipore (Burlington, MA, USA) was used to obtain ultrapure water. Chlorogenic and gallic acid, luteolin, rutin, catechin, hesperidin, and caffeine (>99% HPLC) were from Sigma-Aldrich (St. Louis, MO, USA).

To quantify the phenolic compounds, calibration curves were created by injecting five different concentrations of standard substances dissolved in methanol. The equations of the curves were then used for the quantitative calculation for each phenolic compound. Hydroxycinnamic acids were quantified as the chlorogenic acid equivalent; hydroxybenzoic acids and gingerols as the gallic acid equivalent; flavones as the luteolin equivalent; flavanones as the hesperidin equivalent; flavonols as the rutin equivalent; and flavanols as the catechin equivalent. Caffeine was calculated using the equation of the curve for caffeine. From the specialized literature and the Phenol-Explorer database, we used information to compare the UV-Vis absorption, retention time, and mass spectra of standard compounds to our findings to quantify the phenolic compounds.

### 2.4. In Vitro Antioxidant Activity

#### 2.4.1. DPPH (1,1-Diphenyl 2 picrylhydrazyl) Radical Scavenging Capacity

The *M. recutita* and *Z. officinale* effects were tested against the DPPH radical. First, a sample solution was added to the DPPH methanol solution. The absorbance was measured at 517 nm after 30 min of incubation. The incubation was performed at room temperature in the dark. The result is expressed as IC_50_ (μg/mL). The antioxidant activity is calculated using the following formula: (AA%) = [(OD control − OD sample)/OD control] × 100, where OD represents the optical density, and is expressed in percentages. The result obtained is then converted to Trolox equivalents (TEs), by using the Trolox (TX) calibration curve of standard solutions (0.5–5 μg/mL). The antioxidant potential was interpreted as follows: very good antioxidant potential for the values of IC_50_ < 50 μg TE/mL, good antioxidant potential for the values of IC_50_ 50–100 μg TE/mL, weak antioxidant potential for the values of IC_50_ 100–200 μg TE/mL, and no antioxidant potential for the values of IC_50_ of >200 μg TE/mL [22,23].

#### 2.4.2. Ferric Reducing Antioxidant Power

The ferric reduction antioxidant power was measured using the colorimetric method FRAP, which is based on the ability of antioxidants to reduce the colorless [Fe^3+^-(2,4,6-Tris(2-pyridyl)-s-triazine)2]^3+^ complex to a complex colored in the intensive blue [Fe^2+^-(TPTZ)2]^2+^. This is carried out in an acidic medium [24,25]. The FRAP reagent was made by mixing acetate buffer (0.3 M, pH 3.6), TPTZ (10 mM) in 40 mM HCl, and ferric chloride (20 mM) in a ratio of 10:1:1 (*v*/*v*/*v*). The spectrophotometric measurements were conducted at 593 nm after 30 min of room-temperature incubation. The data obtained are expressed in μg TE/mL [26].

#### 2.4.3. Hydrogen Peroxide Scavenging Activity

The scavenging activity of H_2_O_2_ was evaluated by adding distilled water to the *M. recutita* and *Z. officinale* extract, followed by mixing it with a H_2_O_2_ solution, for 10 min. The absorbance was assessed at 230 nm with a phosphate buffer blank as a reference. The formula used to calculate the quantity of H_2_O_2_ (in percentages) was scavenged H_2_O_2_% = (A control − A sample/A control) × 100. The results are expressed as IC_50_ in μgTE/mL.

#### 2.4.4. Nitric Oxide Radical Scavenging Activity

Next, 0.1% *w*/*v* naphthyl ethylene–diamine–dihydrochloride and the Griess reagent [27] were used to measure the NO radical scavenging activity. For that, *M. recutita* and *Z. officinale* extracts were added to a mixture of phosphate-buffered saline (PBS) and sodium nitroprusside (SNP), at a pH of 7.4. At a temperature of 25 °C, the mixture was then incubated, and then it was combined with sulfanilic acid (0.33% in 20% glacial acetic acid). After 5 min, naphthyl ethylene–diamine–dihydrochloride (0.1% *w*/*v*) was also added. The solution obtained was incubated and vortexed for another 30 min, and the absorbance of the chromophores was measured at 546 nm. The formula used for the percentage inhibition was as follows: NO inhibition % = (A blank − A sample/A blank) × 100, and results are expressed in quercetin equivalent (μg QE/mL).

### 2.5. In Vivo Antioxidant and Anti-Inflammatory Activity

#### 2.5.1. Animal Subjects

Adult male albino Wistar rats (200–250 g ± 15 g) from the “Iuliu Haţieganu” University of Medicine and Pharmacy in Cluj-Napoca’s animal facility were utilized for the conduction of this study. The animals were given as much water as they needed and regular rat pellets (Cantacuzino Institute in Bucharest, Romania). They were held in polypropylene cages at 21 °C, with 12 h/12 h light/dark cycles and an air humidity level of about 55%.

#### 2.5.2. Experimental Protocol

For the experiment, we used 10 groups of rats, which were randomly divided (n = 5): CONTROL group, inflammation (INFL) group, inflammation and Diclofenac (10 mg/kg b.w./d) (DICLO) group, inflammation and Trolox (50 mg/kg b.w./d) (TROLOX) group, 3 inflammation groups with 1mL of the three dilutions of *M. recutita* (M100, M50, M25), and 3 inflammation groups with 1mL of the three dilutions of *Z. officinale* (Z100, Z50, Z25). For the first 7 days, the 6 plant dilutions were administered orally (1 mL/animal). Inflammation was induced on the first day by turpentine oil intramuscular injections (0.6 mL/kg b.w.). All treatments were administered daily by gavage for 7 days. The CONTROL received tap water (1 mL/d). On the eighth day, the rats were anesthetized with ketamine (60 mg/kg body weight) and xylazine (15 mg/kg body weight) [28], and blood was drawn by puncture from the retroorbital sinus and the serum was separated. Then, it was stored at −80 °C until use [29]. Each animal was used only once and then, under general anesthesia, killed by cervical dislocation. The study was approved by the Iuliu Haţieganu University of Medicine and Pharmacy Ethics Committee (nr. 372/2023).

#### 2.5.3. In Vivo Oxidative Stress Markers and Anti-Inflammatory Assessment

As performed in many previous studies, we evaluated general oxidative stress biomarkers [30], like total antioxidant capacity (TAC) [31], total oxidant status (TOS) [32], and the oxidative stress index (OSI) [33], and also some specific biomarkers, such as malondialdehyde (MDA) [34], advanced oxidation protein products (AOPPs) [35], and total thiols (SH) [36]. For the assessment of the anti-inflammatory activity, nitric oxide was measured (NO) [37].

Total antioxidant capacity was measured using the colorimetric approach of a Fenton-type reaction involving the antioxidants from the serum. The outcomes were reported as mmol Trolox equivalent per liter (mmol TE/L).

The total oxidative status was measured using an automated colorimetric method. Oxidants can oxidize the ferrous ions Fe^2+^ complex to ferric ion Fe^3+^, because of their reactivity. An acidic reaction environment was introduced into the blood sample, which contains glycerol molecules. Spectrophotometry can detect the complex formed between the ferric ion and the Xylenol orange. The color intensity of these complexes is directly proportional to the total amount of oxidative molecules. Hydrogen peroxide was used for calibrating the essay. The findings were represented in (μmol H_2_O_2_ Equiv./L).

The oxidative stress index can be calculated mathematically, as it is represented by the ratio between the total oxidative status (TOS) and the antioxidant capacity (TAC) [38]. The formula used was as follows: OSI = TOS / TAC, which indicates a direct proportional relationship between OSI and TOS, and an indirect one between OSI and TAC.

Malondialdehyde is useful for the evaluation of lipid peroxidation [39]. For that, we used serum and trichloroacetic acid (TCA), in the 10% concentration. We also added 5 mM of ethylenediaminetetraacetic acid, 0.5 g/mL butylated hydroxytoluene, and 8% sodium dodecyl sulfate, of which the reaction determined the formation of a solution, which was incubated at room temperature. After the incorporation of 0.6% thiobarbituric acid, for 30 min, the mixture was heated up to 95 °C. At a wavelength of 532 nm, we used spectrophotometry to measure the mixture obtained. MDA is conveyed in serum as nmol/mL.

Advanced oxidation protein products, a biomarker for protein oxidation, were assessed using the method described by Witko-Sarsat et al. Samples of serum and chloramine T (used as a blank) were diluted to 10% in PBS. Glacial acetic acid and then potassium iodide were added. The absorbance of the samples was measured at 340 nm, and the AOPP concentration was expressed in μM chloramine-T equivalents per liter.

Nitric oxide synthesis was determined indirectly by using the Griess reaction which measures total nitrites and nitrates. Briefly, first serum was deproteinized with a 3:1 (*v*/*v*) solution of methanol/diethyl ether. Then, nitrates were subsequently reduced to nitrites by adding vanadium (III) chloride, and then added to the Griess reagent. Sample absorbance was interpreted and noted at 540 nm, and the results were expressed as nitrite µmol/L [40,41].

The total thiols serum concentration can be expressed in mmol GSH/mL after its quantification by using Elmann’s reagent. Of 20 mM tris-HCl buffer pH 8.2, 0.6 mL was added to 0.2 mL of serum, together with 3.16 mL of extra absolute methanol and 0.04 mL of 10 mM 5,5′-dithiobis(2-nitrobenzoic acid) (DTNB) in absolute methanol. After a 15–20 min development period, the solution was centrifuged for 20 min, at 3000× *g*. The supernatant’s absorbance was calculated at 412 nm. A standardized curve was established using solutions with glutathione (GSH) concentrations, in a range from 0.25 to 2 mM.

The spectroscopic measurements of all parameters were carried out by using a Jasco V-530 UV-Vis spectrophotometer (Jasco International Co., Ltd., Tokyo, Japan).

#### 2.5.4. Statistical Analysis

All the obtained values are expressed as the means ± SEM. A one-way variance analysis (ANOVA) and the Bonferroni–Holm post hoc test were used for the comparison of the different groups. The values were considered significant for *p* < 0.05. The statistical analysis was conducted and synthesized by using Microsoft Office Excel 2016, Microsoft Office Excel 2011 for Macintosh, and Daniel’s XL toolbox.

## 3. Results

### 3.1. Phytochemical Analysis

The TPC of *M. recutita* was 3.95 ± 0.12 mg GAE/100 g d.w. of plant material, and the TFC was 288.06±19.55 mg QE/100g d.w. plant material. For *Z. officinale*, the TPC was 4.94 ± 0.42 mg GAE/100 g d.w. of plant material and the TFC was 48.03 ± 7.03 mg QE/100g d.w. of the plant material. The study showed that TPC was higher for ginger, while the TFC was statistically significantly higher for chamomile (Table 1).

### 3.2. High-Performance Liquid Chromatography Coupled with Electrospray Ionization

#### Mass Spectrometry (HPLC-ESI MS) Analysis

**Figure 1:**
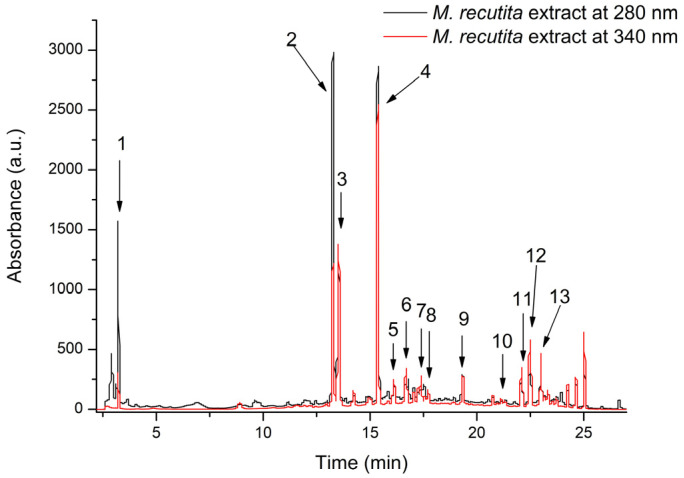
HPLC chromatogram of phenolic compounds from the *M.recutita* extract registered at 280 and 340 nm. The peak identification is provided in Table 2.

In our study, the HPLC-DAD-ESI MS analysis identified substantial concentrations of hydroxybenzoic acid, flavones, and flavonoles for the *M. recutita* extract. To identify the polyphenolic compounds in *M. recutita*, an optimized HPLC/MS method was used for the quantification and identification of the polyphenolic compounds. The phenolic compounds were detected by matching the retention time, UV-Vis absorption spectra, and mass spectra with those of reference standard compounds, with the data from the specialized literature, and from the Phenol-Explorer database. A total of 13 compounds were identified in the *M. recutita* extract, 7 being flavones, 4 flavonols, and 2 being hydroxybenzoic acids. Of the 13 phenolic compounds identified, the flavones subclass represented 53.86% of the phenolic compounds and the flavonol subclass 36.76% (Figure 1, Table 2).

**Figure 2:**
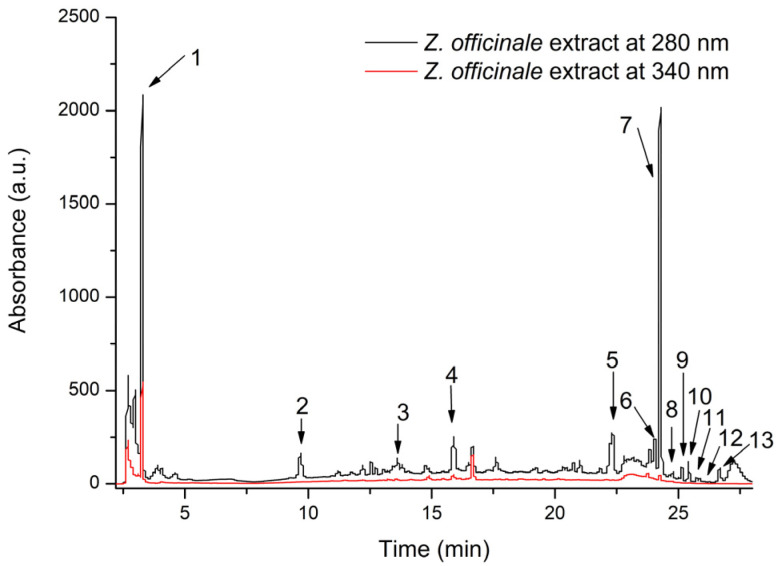
HPLC chromatogram of phenolic compounds from the *Z. officinale* rhizome extract registered at 280 and 340 nm. The peak identification is provided in Table 3.

For *Z. officinale*, a total of 13 compounds were identified, 4 of them being hydroxybenzoic acids (30.76%) and the other 9 being gingerol derivatives (69.23%) (Table 3, Figure 2).

### 3.3. In Vitro Antioxidant Activity

The ethanolic extracts of *M. recutita* and *Z. officinale* exhibited good in vitro antioxidant activity. DPPH radical scavenging capacity, H_2_O_2_ scavenging activity, and FRAP had lower IC_50_ than Trolox (*p* < 0.01), and NO radical scavenging activity was smaller than that of quercetin (*p* < 0.05). The comparison of in vitro antioxidant activity between *M. recutita* and *Z. officinale* was not statistically significant (*p* > 0.05) (Table 4).

### 3.4. In Vivo Antioxidant Activity

The in vivo antioxidant activity of the *M. recutita and Z. officinale* extracts was evaluated using specific and global biomarkers (Table 5).

The oxidative stress index was significantly increased (*p* < 0.001) for the INFLAM group when compared to the CONTROL group because TOS had higher values and TAC had lower values. Moreover, when compared to the CONTROL group, the INFLAM group had an increased value of MDA (*p* < 0.01), NO (*p* < 0.001), and AOPP (*p* < 0.01), and an associated lower SH (*p* < 0.01).

The diclofenac antioxidant activity produced a moderate decrease (*p* < 0.01) in TOS, OSI, MDA, NO, and AOPP, when the values were compared to the INFLAM group. TAC and SH were not influenced by diclofenac (*p* > 0.05).

Trolox administration produced a moderate decrease (*p <* 0.01) in TOS, OSI, MDA, NO, and AOPP levels, plus an increase in TAC (*p <* 0.01) and SH (*p* < 0.001). When compared to the DICLO group, trolox effect produced a more significant increase in TAC and SH levels (*p* < 0.01), and a more important decrease in TOS, OSI, MDA, and AOPP levels (*p* < 0.05).

For *Z. officinale*, only ZO50% and ZO25% determined a small increase in TAC (*p* < 0.05) and a very significant increase in SH (*p* < 0.001). At the same time, *Z. officinale* caused a moderate reduction in TOS, OSI, and MDA (*p* < 0.01). NO levels were also reduced, but ZO100% had a smaller efficiency (*p* < 0.01) than ZO50% and ZO25% (*p* < 0.001). *Z. officinale* did not influence AOPP (p>0.05).

When compared to the diclofenac’s effect, *Z. officinale* administration had similar efficiency on TOS, OSI, NO, and MDA (*p* < 0.05), but regarding AOPP, diclofenac was a better inhibitor (*p* < 0.001). The antioxidant markers levels, respectively, TAC (*p* < 0.05) and SH (*p* < 0.001), were increased more by *Z. officinale* extract than by diclofenac.

Between *Z. officinale* and trolox effects, there were no noticeable differences upon TOS, OSI, MDA, NO, and SH (*p* < 0.05). For TAC and AOPP, trolox had a better activity (*p* < 0.01).

The *M. recutita* extract administration significantly reduced the TOS, OSI, and NO (*p* < 0.01), and MDA was moderately lowered (*p* < 0.05). At the same time, *M. recutita* extract MR50% and MR25% increased TAC (*p* < 0.01). AOPP and SH were not influenced by the M. recutita extract.

When compared to diclofenac, *M. recutita* extract MR50% and MR25% caused a better increase in TAC, but a smaller increase in SH levels. For TOS and OSI, *M. recutita* extract had a better inhibitory activity than diclofenac (*p* < 0.05), but for MDA, NO, and AOPP levels, *M. recutita* extract had a lower inhibitory effect (*p* < 0.05). When compared to trolox, *M. recutita* extract had a lower effect on TAC (*p* < 0.01) and SH (*p* < 0.001). *M. recutita* extract had a better inhibitory activity on TOS and OSI (*p* < 0.01) than trolox, but for MDA, NO, and AOPP, trolox caused a more significant reduction than the *M. recutita* extract (*p* < 0.01).

### 3.5. PCA Analysis

**Figure 3:**
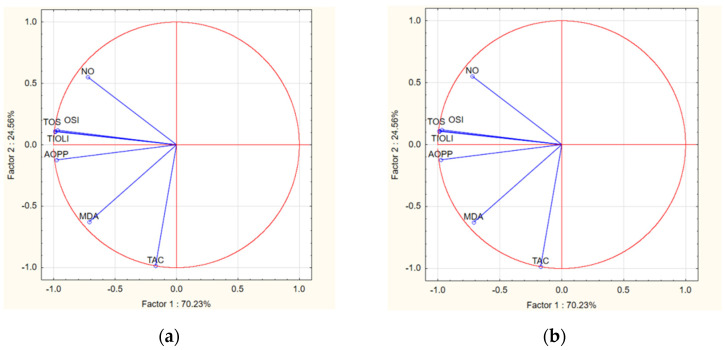

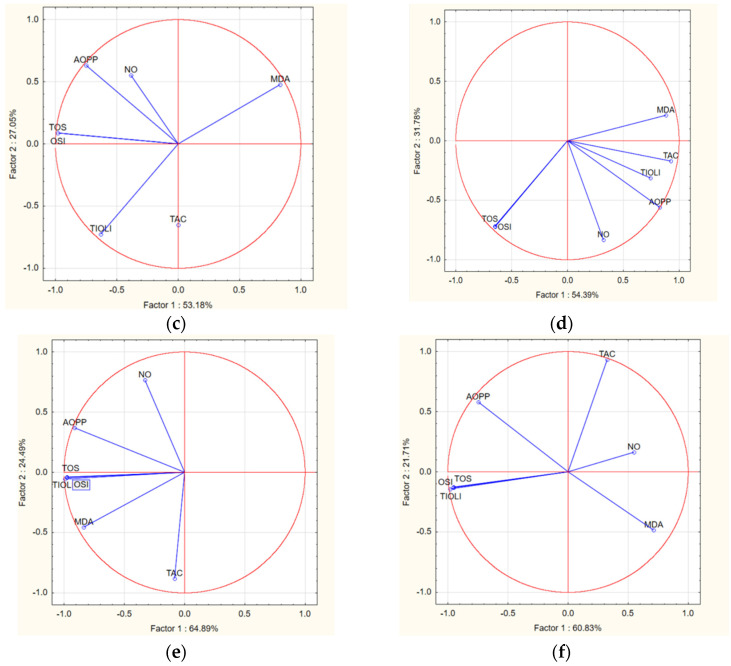
Oxidative stress markers of PCA analysis in groups treated with *Z. officinale* and *M. recutita* extracts. (**a**) ZO100%—*Z. officinale* extract 100% treatment; (**b**) ZO50%—*Z. officinale* extract 50% treatment; (**c**) ZO25%—*Z. officinale* extract 25% treatment; (**d**) MR100%—*M. recutita* extract 100% treatment; (**e**) MR50%—*M. recutita* extract 50% treatment; (**f**) MR25%—*M. recutita* extract 25% treatment.

To establish if there are correlations between the oxidative stress markers that were analyzed after the treatments with *Z. officinale* and *M. recutita* extracts, we performed a PCA analysis (Figure 3).

For the ZO100%, the PCA analysis showed good, positive correlations between TOS, OSI, MDA, NO, and AOPP, but TAC and SH were not correlated. In ZO50%, from the oxidants, only TOS, OSI, AOPP, and NO were positively correlated, and all these markers were negatively correlated with MDA. TAC was positively correlated with SH. In the group, ZO25% TOS, OSI, MDA, and AOPP were positively correlated, and NO was only positively correlated with TOS, OSI, and AOPP. There was a small positive correlation between TAC and SH.

For the MR100%, a significant positive correlation was found between TOS, OSI, MDA, and AOPP, while NO was positively correlated only with MDA. Between TAC and SH, there was a moderate negative correlation. In the MR50% group, the PCA analysis showed a good positive correlation between TOS, OSI, and NO, between NO and AOPP, and between MDA and AOPP. The TAC and SH were positively correlated in this treatment. MR25% showed a significant correlation between TOS, OSI, and AOPP, with NO and MDA being negatively correlated with TOS, OSI, and AOPP. TAC and SH were negatively correlated.

## 4. Discussions

This study aimed to explore the antioxidant and anti-inflammatory effects of *M. recutita* and *Z. officinale,* and contribute to the current literature on the potential of these plants as an anti-inflammatory herbal medicine.

As explored in previous studies, herbal medicine may develop to provide a comparative alternative to nonsteroidal anti-inflammatory drugs, avoiding their side effects. The applications of phytotherapy in medicine are extensive. Historically, plant extracts have been made from most parts of plants, being used as preventive or curative measures for many diseases, such as atherosclerosis, hypertension, dementia, diabetes, cancer, or neurodegenerative diseases. They have also been known to protect against cell aging and oxidative stress-induced diseases, or for their ability to improve cognitive function [42,43].

The anti-inflammatory and antioxidant effects of a plant extract are correlated to the phytochemical characteristics of the plant [44]. The main bioactive compounds found in plant extracts are polyphenols, which can scavenge free radicals by single electron transfer or hydrogen atom transfer mechanisms [44]. In this study, the total polyphenol content was determined using the Folin–Ciocâlteu method. In previous studies, the composition of the extracts was shown to vary depending on the geographic area, the part of the plant, or the method used for the extraction [45]. In this study, the extracts were obtained from different plant parts, respectively, the blossoms for *M. recutita*, and the rhizomes of *Z. officinale*. The results showed that the TPC content of the two extracts is significant, being higher for *Z. officinale* than for *M. recutita.*

From the natural compounds found in plants, flavonoids are known to provide taste and fragrance to fruits, seeds, and flowers [46]. They are non-volatile substances with an important role in the plant’s medicinal effect. They were evaluated by measuring the TFC, which is shown to be also higher for *M. recutita* than for *Z. officinale*.

Since HPLC-DAD analysis is one of the most used analytical techniques to separate, identify, and quantify the components of a mixture [47], we used it to identify and assess the phenolic compounds of *M. recutita* and *Z. officinale* extracts. For *M. recutita*, our study identified high concentrations of hydroxybenzoic acids, flavones, and flavonols. Of the 13 components found in *M. recutita’s* extract, 11 were flavonoids, representing the most significant percentage, although some of them did not have high concentrations. The most important phenolic compounds found were 2,3-dihydroxybenzoic acid, apigenin-diglucoside, apigenin-glucoside, and apigenin-acetyl-glucoside. In another study, published in 2020, Nefeli S. et al. also found thirteen phenolic compounds in *M. recutita*, but using aqueous extracts from the plant [48]. The identification of the above compounds in the studied plant extract confirms the antioxidant potential, as has been demonstrated numerous times before, in the specialized literature.

In the *Z. officinale* extract, the HPLC-DAD analysis also identified 13 compounds, the highest values being for 6-gingerol and 2,3-dihydroxybenzoic acid. Most of the compounds found in this extract were gingerol derivates (9 out of 13), and hydroxybenzoic acids, which are also known for their antioxidant activity, as demonstrated in a study from 2021, conducted by Monika K. et. al. [49]. This is consistent with another study performed by Sara H.H.A et al. in 2023 [50]. The same study demonstrated the antioxidant effect of *Z. officinale*.

The inhibitory effect on the oxidation reaction appears due to the antioxidant activity of an extract. The *Z. officinale* and *M. recutita* antioxidant effect was suggested by their phytochemical composition. First, the in vitro antioxidant activity was assessed by measuring the DPPH radical scavenging capacity, the ferric reducing antioxidant power, the scavenging activity of H_2_O_2_, and the radical scavenging activity of nitric oxide. Both extracts showed good antioxidant activity when tested in vitro. In comparison to Trolox, DPPH, and H_2_O_2_ had lower IC_50_, and NO radical scavenging activity was smaller than that of quercetin.

Even though the composition of the tested plant extracts and their in vitro activity showed good antioxidant effects, we further conducted an in vivo experiment, to confirm our findings and to assess the antioxidant activity by measuring specific and global biomarkers. To assess the in vivo antioxidant effect of *M. recutita* and *Z. officinale* rhizome extract, we used animals with turpentine oil-induced acute inflammation, and we compared the effects of diclofenac and trolox treatment, against those of our extracts.

Trolox is a synthetic analog of alpha-tocopherol, that suppresses the NF-kB signaling, activates Nrf2, and inhibits matrix metalloproteinases. Its effect was studied on arthritis and other inflammatory diseases, and it concluded that it lowers the serum levels of many pro-inflammatory mediators, as shown by Shaaban H.H. et al. in 2022 [51]. Diclofenac is a nonsteroidal anti-inflammatory drug that also has antipyretic and analgesic properties, being effective in many acute and chronic inflammatory conditions. It acts by inhibiting the cyclooxygenase-1 (COX-1) and cyclooxygenase-2 (COX-2), but also inhibits lipoxygenase enzymes, and activates the nitric oxide-cGMP antinociceptive pathway, as shown in the article published by J. G. Tong in 2010 [52]. This drug was studied extensively, and it is commonly used so we can assume that a comparison with its effects could be considered very relevant.

Ginger and chamomile are two of the most commonly used plants in alternative medicine. Studies have shown that almost 80% of the population tends to use traditional medicine as primary health care, but herbal extracts and their active components are often required as adjuvant for this therapy, especially for preventive measures [53].

Oxidative stress occurs in many diseases and arises when the body’s scavenging mechanisms and antioxidative capacity are unable to neutralize the effects of oxidants, leading to oxidative damage. So, oxidative stress biomarkers are indicators of the severity of an illness. Oxygen reactive species (ROS) represent the highest percent of the active oxides and have been proven to be involved in the development of malignant tumors, through the inhibition of their cell’s apoptosis, together with their sustained proliferation, causing DNA damage and genetic mutations, malignant cells invasion and metastasis [54]. So, the general test of oxidative stress TOS, and OSI, had higher levels in the presence of inflammation. The drug therapy should induce the decrease of these parameters. Diclofenac and Trolox produced a moderate decrease in TOS and OSI, and our study showed that *Z. officinale* also determined a moderate reduction of the same parameters. As for *M. recutita*, the effect on TOS and OSI was better than that of diclofenac and trolox. These results sustain the idea that the plant extracts we studied have good antioxidant properties if used as an adjuvant treatment.

While TOS and OSI should be reduced in the presence of antioxidant therapy, the TAC should increase. Our results showed that TAC wasn’t influenced by Diclofenac, and Trolox administration produced an increase in TAC. Only the 25% and 50% dilution of *Z. officinale* determined a small increase in TAC, thus having a better effect than traditional medicines. MR50% and MR25% also caused a better increase of TAC if compared to Diclofenac, but a smaller effect compared to Trolox. Our results are similar to other recent studies, as Miraj S. et al. have shown in 2016 [12], or as Iswaibah M. et al. published in 2023 [55].

Malondialdehyde is a result of fatty acids peroxidation, being used as a marker of oxidative stress in many diseases that involve oxidative stress. It is the most extensively studied compound of lipid peroxidation, because of its many toxic and mutagenic effects, according to the study published by Cordiano R. Et.al in 2023, following the study of Esterbauer H. et. al. in 1990 [56,57]. In 2022, Ballester P. et al. showed the effects of ginger in lowering MDA levels [58] as did Javadi I. et al. in 2015, about chamomile [59]. Similar to other studies found in specialized literature, we have also shown that *Z. officinale* caused a moderate reduction in MDA levels. Its efficiency was almost the same when compared to that of Diclofenac and Trolox. On the other hand, *M. recutita* extracts showed moderate inhibitory effects on MDA levels, but lower than that of diclofenac and trolox. In the case of *M. recutita*, only MR100% caused a correlated decrease in MDA, TOS, and OSI. MDA changes suggested that MDA had an important contribution to *Z. officinale* and *M. recutita* ethanol extracts’ antioxidant activity.

Inflammation facilitates inducible isoform of this enzyme (iNOS) activation and excessive NO synthesis [60]. In turn, NO can react with superoxide and produce reactive nitrogen species (RNS). Then, ROS and RNS enhance inflammatory processes and the damaging effect on lipids, proteins, and DNA, with potential pathological consequences [61]. Our study showed that in vitro, *Z. officinale* reduces NO formations, and the in vivo effect was dependent on the dilution of the extract, having the most statistically significant result. Furthermore, the ZO 100% and ZO 50% effect on NO was correlated with TOS and OSI reduction. *Z. officinale* effects were similar to Diclofenac’s and Trolox’s. The *M. recutita* extract also reduced NO formation but had a lower effect than Diclofenac and Trolox. For MR100% and MR50% NO lowering effect was correlated with TOS and OSI decrease. Taken together, these results indicated that NO had also an important contribution to *Z. officinale* and *M. recutita* antioxidant activities.

AOPPs are the result of the reaction between proteins and chlorinated oxidants (HOCl/OCl^-^) and are markers of systemic oxidative stress [62]. At the same time, AOPP are involved in the regulation of cell functions and may be involved in the inflammatory response as proinflammatory compounds [63], because AOPP can increase ROS production [64]. In the present study, AOPP was not influenced by the administration of *Z. officinale* and *M. recutita* extract.

We also assessed the total thiols, as markers of the inflammatory response. Thiol reduction due to their irreversible oxidation caused by high ROS concentration can be a marker of the loss of antioxidant plasma proteins [65]. While Diclofenac did not have an important influence on SH levels, Trolox did show a significant increase. Our study provides evidence that SH levels were significantly increased by *Z. officinale* treatment, but *M. recutita* did not have a statistically significant effect on the SH.

From the current data, and consistent with other studies, the current study showed that both *Z. officinale* and *M. recutita* ethanol extracts have a positive influence by reducing inflammation-induced oxidative stress, and the efficiency can be compared to the effects of drugs, such as trolox and diclofenac. The effects were dose-dependent, the lower concentrations being more efficient. Taking together, both *Z. officinale* and *M. recutita* have the potential to be good candidates for the discovery of new anti-inflammatory treatments targeting oxidative stress.

## 5. Conclusions

Treatment with *Z. officinale* and *M. recutita* alleviated inflammation-induced oxidative stress. Based on the information available, we found no other comparative study that specifically examines the antioxidant activity of the ethanol extracts of *Z. officinale* and *M. recutita*. The data reported in this article provide evidence that the antioxidant activity mechanism of *M. recutita* ethanol extract primarily involves enhancing antioxidant levels, while for *Z. officinale* the mechanism combines an increase in antioxidants with a reduction in oxidants. These findings suggest that *M. recutita* and *Z. officinale* extracts could serve as promising adjuvant antioxidant therapies for managing inflammatory diseases. Further studies are necessary to confirm these results and explore their therapeutic potential.

## Figures and Tables

**Table 1:** Total polyphenol content and total flavonoid content of *M. recutita* and *Z. officinale* ethanolic extracts.

Plant Extract	Total Polyphenols Content (mg GAE/g d.w. Plant Material)	Total Flavonoids Content (mg QE/100 g d.w. Plant Material)
*M. recutita*	3.95 ± 0.12	288.06 ± 19.55
*Z. officinale*	4.94 ± 0.42	48.03 ± 7.03

**Table 2:** Phenolic compound identification and quantification in the *M. recutita* ethanolic extract.

Peak No.	Rt (min)	UV λmax (nm)	[M+H]^+^ (m/z)	Compound	Subclass	*M. recutita* Compounds Concentration (μg/mL)
1	3.22	270	155	2,3-Dihydroxybenzoic acid	Hydroxybenzoic acid	166.676
2	13.25	340, 245	595, 271	Apigenin-diglucoside	Flavone	105.391
3	13.52	340, 245	433, 271	Apigenin-glucoside	Flavone	137.051
4	15.36	341, 245	475, 271	Apigenin-acetyl-glucoside	Flavone	210.275
5	16.12	360, 250	493, 331	Quercetin-dimethyl eter-glucoside	Flavonol	86.125
6	16.68	340, 245	565, 271	Apigenin-glucoside-arabinoside	Flavone	48.113
7	17.38	350, 260	479, 317	Isorhamnetin-glucoside	Flavonol	66.597
8	17.71	350, 265	463, 287	Kaempferol-glucuronide	Flavonol	48.256
9	19.33	290	213, 199	Methyl-syringic acid	Hydroxybenzoic acid	98.630
10	21.31	360, 250	303	Quercetin	Flavonol	37.378
11	22.08	340, 245	417, 271	Apigenin-rhamnoside	Flavone	39.254
12	22.48	340, 250	285	Methylapigenine	Flavone	72.889
13	22.99	340, 245	271	Apigenin	Flavone	41.577
				Total phenolics		1158.210

**Table 3:** Identification and quantification of phenolic compounds in the *Z. officinale* rhizome ethanolic extract.

Peak No.	Rt (min)	UV λmax (nm)	[M+H]^+^ (m/z)	Compound	Subclass	*Z. Officinale* Compounds Concentration (μg/mL)
1	3.26	270	155	2,3-Dihydroxybenzoic acid	Hydroxybenzoic acid	220.715
2	9.68	280	155	Protocatechuic acid	Hydroxybenzoic acid	68.116
3	13.61	280	169	Vanillic acid	Hydroxybenzoic acid	36.353
4	15.89	280	153	Vanillin	Hydroxybenzoic acid	96.905
5	22.34	280	353	Diacetoxy-4-gingerdiol	Gingerol derivative	68.770
6	24.05	220, 280	297	6-Gingerdiol	Gingerol derivative	56.160
7	24.25	231, 280	295	6-Gingerol	Gingerol derivative	325.521
8	24.78	229, 279	309	Methyl-6-gingerol	Gingerol derivative	12.798
9	25.14	221, 281	323	8-Gingerol	Gingerol derivative	13.720
10	25.41	226, 281	277	6-Shogaol	Gingerol derivative	17.675
11	25.73	220, 254, 370	291	1-Dehydro-6-gingerdione	Gingerol derivative	3.727
12	26.18	220, 281	351	10-Gingerol	Gingerol derivative	6.642
13	26.66	229, 279	395	Methyl diacetoxy-6-gingerdiol	Gingerol derivative	17.140
				Total phenolics		944.241

**Table 4:** In vitro antioxidant activity of the *M. recutita* and *Z. officinale* ethanolic extracts.

Sample	DPPH (μg TE/mL)	NO (μgQE/mL)	H_2_O_2_ (μg TE/mL)	FRAP (μg TE/mL)
*Z. officinale* IC_50_	111.5 ± 9.06	54.83 ± 3.41	65.64 ± 7.05	82.75 ± 5.56
*M. recutita* IC_50_	108.97 ± 10.12	58.64 ± 2.72	59.96 ± 4.33	88.22 ± 7.05
Trolox IC_50_	11.2 ± 0.98	-	24.23 ± 1.92	11.077 ± 0.96
Quercetin IC_50_	-	20.58±0.87	-	-

DPPH—α,α-diphenyl-β-picrylhydrazyl; NO—nitric oxide; H_2_O_2_—hydrogen peroxide; FRAP—ferric reducing antioxidant power; QE—quercetin equivalent; TE—trolox equivalent.

**Table 5:** Serum oxidative stress markers of the rats with turpentine oil-induced inflammation treated with *Z. officinale* and *M. recutita* extract.

GROUP	TAC (mmol TE/L)	TOS (µmol H_2_O_2_E/L)	OSI	MDA (nmol/L)	NO (µmol/L)	AOPP (µmol/L)	SH (µmol/L)
CONTROL	1.11 ± 0.00	27.56 ± 4.35	24.81 ± 3.91	3.61 ± 0.46	22.94 ± 3.49	25.75 ± 2.11	458.20 ± 94.95
INFLAM	1.1075 ± 0.00 **	45.68 ± 5.50 ***	41.24 ± 5.73 ***	5.80 ± 1.13 **	71.82 ± 11.16 ***	55.48 ± 3.75 **	299.40 ± 53.72 **
DICLO	1.1074 ± 0.00	35.70 ± 3.42 ##	32.23 ± 3.06 ##	3.15 ± 0.35 ##	49.15 ± 8.29 ##	34.29 ± 3.65 ##	356.20 ± 72.07 #
TX	1.1107 ± 0.00 ##	29.32 ± 5.50 ##	26.40 ± 4.94 ##	2.71 ± 0.30 ##	52.94 ± 5.20 ##	23.25 ± 0.89 ###	412.50 ± 45.35 ###
ZO100%	1.1089 ± 0.00	32.86 ± 8.31 ##	29.63 ± 7.49 ##	3.90 ± 0.35 ##	56.80 ± 7.14 ##	57.86 ± 12.62	468.60 ± 63.45 ###
ZO50%	1.1102 ± 0.00 #	28.98 ± 4.26 ##	26.10 ± 3.67 ##	3.51 ± 0.22 ##	46.41 ± 3.28 ###	53.97 ± 5.97	436.60 ± 74.65 ###
ZO25%	1.1100 ± 0.00 #	35.90 ± 10.03 ##	32.34 ± 9.03 ##	3.85 ± 0.61 ##	45.22 ± 4.54 ###	52.51 ± 15.72	466.20 ± 72.52 ###
MR100%	1.1085 ± 0.00	25.71 ± 3.09 ###	23.19 ± 2.79 ###	4.30 ± 0.45 #	56.06 ± 7.22 ##	52.56 ± 5.94	292.60 ± 48.02
MR50%	1.1101 ± 0.00 #	24.55 ± 4.47 ###	22.09 ± 4.03 ###	4.21 ± 0.10 #	58.28 ± 7.67 #	57.37 ± 13.18	309.80 ± 106.23
MR25%	1.1113 ± 0.00 ##	30.28 ± 4.05 ##	27.28 ± 3.67 ##	4.36 ± 0.29 #	60.50 ± 3.78 #	51.10 ± 7.00	303.80 ± 87.17

Data are presented as the mean ± SD. Abbreviations: TAC—total antioxidant capacity; TOS—total oxidative status; OSI—oxidative stress index; MDA—malondialdehyde; NO—nitrites and nitrates; AOPP—advanced oxidation protein products; SH—total thiols; TX—trolox; INFLAM—inflammation group; DICLO—inflammation treated with diclofenac; TE—trolox equivalent; H_2_O_2_E—hydrogen peroxide equivalent; ZO100%—*Z. Officinale* extract 100% treatment; ZO50%—*Z. officinale* extract 50% treatment; ZO25%—*Z. Officinale* extract 25% treatment; MR100%—*M. Recutita* extract 100% treatment; MR50%—*M. Recutita* extract 50% treatment; MR25%—*M. Recutita* extract 25% treatment. Statistical significance: * vs. control: ** *p* < 0.01; *** *p* < 0.001; # vs. inflammation: # *p* < 0.05; ## *p* < 0.01; ### *p* < 0.001.

## Data Availability

Data are available on request.
